# The Role of Two Linear β-Glucans Activated by c-di-GMP in *Rhizobium etli* CFN42

**DOI:** 10.3390/biology11091364

**Published:** 2022-09-17

**Authors:** Daniel Pérez-Mendoza, Lorena Romero-Jiménez, Miguel Ángel Rodríguez-Carvajal, María J. Lorite, Socorro Muñoz, Adela Olmedilla, Juan Sanjuán

**Affiliations:** 1 Department of Soil and Plant Microbiology, Estación Experimental del Zaidín, CSIC, 18008 Granada, Spain; 2 Department of Organic Chemistry, Faculty of Chemistry, Universidad de Sevilla, 41001 Sevilla, Spain; 3 Department of Stress, Development and Signaling in Plants, CSIC, 18008 Granada, Spain

**Keywords:** β-glucan, *Rhizobium etli*, cyclic-di-GMP, cellulose, mixed-linkage-β-glucan (MLG), symbiosis

## Abstract

**Simple Summary:**

Bacterial exopolysaccharides (EPS) are secreted biopolymers with often critical roles in bacterial physiology and ecology. In addition to their biological role, there is increasing interest for EPS in various industrial sectors. β-glucans are among the most important ones including cellulose as the most abundant organic polymer on earth, but also newcomers, such as the bacterial Mixed Linkage β-Glucan (MLG), displaying a unique repeating unit suggestive of biotechnological potential. In this work we describe *Rhizobium etli* as the first bacterium reported to be able to produce these two linear β-glucans cellulose and MLG. *Rhizobium etli* is an agronomic relevant rhizobacteria able to perform Biological Nitrogen Fixation (BNF) in a symbiotic association with common bean plants. The production and regulation of cellulose and MLG by *Rhizobium etli* CFN42 is discussed and their impact on its free-living and symbiotic lifestyles evaluated.

**Abstract:**

Bacterial exopolysaccharides (EPS) have been implicated in a variety of functions that assist in bacterial survival, colonization, and host–microbe interactions. Among them, bacterial linear β-glucans are polysaccharides formed by D-glucose units linked by β-glycosidic bonds, which include curdlan, cellulose, and the new described Mixed Linkage β-Glucan (MLG). Bis-(3′,5′)-cyclic dimeric guanosine monophosphate (c-di-GMP) is a universal bacterial second messenger that usually promote EPS production. Here, we report *Rhizobium etli* as the first bacterium capable of producing cellulose and MLG. Significant amounts of these two β-glucans are not produced under free-living laboratory conditions, but their production is triggered upon elevation of intracellular c-di-GMP levels, both contributing to Congo red (CR^+^) and Calcofluor (CF^+^) phenotypes. Cellulose turned out to be more relevant for free-living phenotypes promoting flocculation and biofilm formation under high c-di-GMP conditions. None of these two EPS are essential for attachment to roots of *Phaseolus vulgaris*, neither for nodulation nor for symbiotic nitrogen fixation. However, both β-glucans separately contribute to the fitness of interaction between *R. etli* and its host. Overproduction of these β-glucans, particularly cellulose, appears detrimental for symbiosis. This indicates that their activation by c-di-GMP must be strictly regulated in time and space and should be controlled by different, yet unknown, regulatory pathways.

## 1. Introduction

Bacterial β-glucans are polysaccharides with D-glucose units as the only constituent sugar linked by β-glycosidic bonds. Linear unbranched β-glucans include bacterial cellulose (β 1→4 D-glucose), curdlan (β 1→3 D-glucose), and the recently discovered bacterial Mixed-Linkage β-glucan (MLG; (β 1→3;1→4) D-glucose). Other β-glucans are Osmoregulated Periplasmic Glucans (OPG), including Cyclic Glucans, which are universally distributed and their presence is essential for almost all proteobacteria [1]. These polymers occur also as complexes with other polysaccharides and proteins and have been implicated in a variety of roles that assist in bacterial survival, colonization, and cell–cell interactions. The main roles include: mediation of cell adhesion leading to biofilm formation and prevention of desiccation in low-moisture environments due to the water-binding properties of the β-glucans; they are major constituents of capsular materials and enhanced survival in stressful environments, and they may serve as carbohydrate storage and modulators of host defenses enabling infection [2]. 

Bacterial β-glucans are structurally related to others described in eukaryotes, such as cellulose, paramylon, lichenan, pachyman, chromistan and fungal laminarins and mucilage glucans [3]. Cellulose is the most abundant organic polymer on earth and the main structural component of the primary cell wall of most plants, many forms of algae and oomycetes. Cellulose is also produced by certain types of bacteria, including strains of *Komagataeibacter*, *Sarcina* and different genera from Rhizobiaceae family among others. Curdlan is a neutral, essentially linear (1→3)-β-glucan first detected in *Agrobacterium* biovar. 1 (formerly *Alcaligenes faecalis*; ref. [4]) with unique rheological and thermal gelling properties, with applications in the food industry and other sectors. Mixed linkage (1→3;1→4) β-glucans (MLG) have been described as hemicelluloses in Poales (cereals and grasses) but are also found in some embryophytes and lichens (lichenan). Eukaryotic MLGs consist of unsubstituted linear molecules containing distinctive ratios of cello-oligosaccharides (β 1→4) of 3, 4, or higher degrees of polymerization, linked by (β 1→3) bonds [5,6]. We recently described a bacterial MLG that is unique in two aspects: (i) it is the first and so far the only MLG reported in bacteria and (ii) it contains a perfect alternation of β (1→3) and β(1→4) bonds (→3)-β-d-Glc*p*-(1–4)-β-d-Glc*p*-(1→) [7]. The unique repeating unit is expected to deeply affect its rheology and physicochemical behavior compared to others that are better known, such as curdlan, cellulose, and other eukaryotic MLG.

Bis-(3′,5′)-cyclic dimeric guanosine monophosphate (c-di-GMP) is a universal bacterial second messenger. Initially identified as an allosteric activator of bacterial cellulose synthase [8], this second messenger can regulate a wide range of cellular processes, including cell–cell signaling, cell cycle progression, and virulence. Cyclic-di-GMP signaling systems could be classified in four major components: (i) diguanylate cyclases (DGCs, synthesize c-di-GMP from two GTP molecules), (ii) phosphodiesterases (PDEs, degrade c-di-GMP), and (iii) c-di-GMP binding effectors that interact with (iv) target elements to produce a molecular output [9,10]. Its involvement in the bacterial decision to attach to a surface and form a biofilm community makes this molecule a key activator for the production and secretion of different biofilm matrix components, including many exopolysaccharides (EPS) [11]. Up to date more than a dozen EPS are known to be regulated by the bacterial second messenger c-di-GMP, including different β-glucans [12]. 

Rhizobia are soil-dwelling micro-organism that have to constantly face changes of the conditions and rapidly adapt their behavior in order to maximize their fitness in the environment. Bacterial signal transduction is a key system for generating the physiological, genetic, and cellular adaptive responses required at all times. This is even more relevant for bacteria that interact with eukaryotic hosts. The association with the legume plant entail a new and interesting ecological niche for rhizobia, but at the same time the requirement of an accurate regulated genetic program for a successful transition from a saprophytic life in the soil to an intimate association with the plant host. Cyclic-di-GMP turned out to be an important molecule in the regulation of this life-style transition [13]. Due to this symbiotic association with legumes, rhizobia are able to perform an indispensable biological process, biological nitrogen fixation (BNF). BNF depends on the ability of rhizobia to fix nitrogen from the air using the carbon and energy sources provided by the host. The process occurs after the attachment, colonization and infection of the roots, that leads to formation of a novel organ in the legume root, the nitrogen-fixing nodule. BNF is catalyzed by the nitrogenase, a bacterial enzyme that requires micro-oxic conditions and a high energy supply, conditions found in the legume nodule [14]. 

The knowledge on c-di-GMP signaling pathways remains largely fragmentary in plant-associated bacteria. In rhizobia, it has mainly been focused in *Sinorhizobium meliloti* and the symbiotic interaction with its *Medicago* host plant. A gene deletion analysis in all active DGCs of Rm2011 revealed that the resulting c-di-GMP^0^ strain displayed attenuated growth in acidic conditions. However, no further free-living or symbiotic defects were found [15]. Nonetheless, c-di-GMP has been reported to influence production of up to five different EPS in this bacterium [13]. EPS are usually relevant in early the stages of the interaction with the host plant, where surface polysaccharides play a role in the recognition, the surface attachment and the biofilm formation required for the infection process. In *S. meliloti*, c-di-GMP stimulates production of an arabinose-containing polysaccharide (APS), an adhesion polysaccharide of unknown composition (UPP) and the recently discovered β-glucan MLG [7,15,16]. Cyclic-di-GMP activates MLG production through its specific binding to a novel cytoplasmic C-terminal domain of BgsA, the glycosyltransferase (GT) involved in its polymerisation [17]. Cyclic-di-GMP activates APS through its binding to CuxR, an AraC-type transcription activator required for the expression of the APS biosynthetic operon [16]. In addition, c-di-GMP also influences the production of the two symbiotically relevant EPS, activating the production of succinoglycan (EPS I) and negatively regulating the production of galactoglucan (EPS II) at transcriptional level [15]. The APS, UPP, and MLG facilitate cell aggregation, biofilm formation, and surface attachment to the host plant [7,15,16]. 

Cyclic-di-GMP signaling pathways are much less known in *R. etli*. Nonetheless, up to 39 c-di-GMP-related proteins have been predicted in *R. etli* CFN42 (Ret), suggesting an important role of this second messenger in this *Phaseolus vulgaris* symbiont [18]. An artificial increase of c-di-GMP levels upon DGC overexpression in Ret favored the early stages of the interaction with the host plant, but resulted in decreased symbiotic efficiency as plant growth and nitrogen contents were reduced [19]. In contrast to *S. meliloti* reference strains Rm2011 and 1021, Ret is able to produce cellulose. Indeed, the production of this β-glucan was enhanced by c-di-GMP in this strain, promoting biofilm formation and attachment to bean roots [19]. 

Here we demonstrate that Ret is also able produce another c-di-GMP regulated β-glucan, the MLG. The importance of these two β-glucans in free-living and symbiotic lifestyles under physiological and high c-di-GMP conditions are discussed.

## 2. Materials and Methods

### 2.1. Bacteria and Culture Conditions

Bacterial strains and plasmids used in this work are listed in Table 1. *Escherichia coli* was routinely grown in Luria–Bertani broth (LB; containing 10 g/L tryptone, 5 g/L yeast extract, 5 g/L NaCl) at 37 °C. Starting cultures of *Rizobium etli* were grown overnight at 28 °C on TY broth (tryptone-yeast extract-CaCl_2_ [20]) or minimal medium MM [21]. When required, antibiotics and other compounds were added at the following final concentrations: Tetracycline (Tc), 10 μg mL^−1^ (5 μg mL^−1^ for Ret); Congo Red (CR), 50 μg mL^−1^, Calcofluor (CF) 200 μg mL^−1^ (in solid media) or 100 μg mL^−1^ (in liquid media). 

### 2.2. Recombinant DNA Techniques

Molecular biology techniques were performed according to standard protocols and manufacturers’ instructions. Mini-Tn7 transposons were introduced in *R. etli* by bacterial conjugation using the *E. coli* β2163 donor strain [22] in matings as previously described [23].

### 2.3. Construction of bgsA (RHE_PE00363) Mutant and bgsA celAB Double Mutant

To generate a *R. etli* CFN42 derivative carrying a deletion of MLG synthase *bgsA* gene, two fragments flanking the ORF were amplified separately, using two pairs of primers 363-1/363-2 (939 bp) and 363-3/363-4 (808 bp, Table S1). These two fragments were purified and used as template DNA for an overlapping PCR with primers 3631 and 363-4. The final fragment (1,7 Kb) was cloned into pCR^®^-2.1-TOPO^®^ (Invitrogen, Waltham, MA, USA) and sequenced generating TOPOΔbgsA. A correct *Xba*I/*Hind*III DNA insert was isolated and subcloned into the suicide plasmid pK18*mobSacB* [24] generating the pK18ΔbgsA. The pK18ΔbgsA plasmid was introduced into *R. etli* CFN42 by a biparental conjugation from *E. coli* S17.1 strain, and the *bgsA* deletion of 1425 bp was generated by homologous recombination, following procedures described in [24]. The Ret ΔbgsA mutant contained an untagged (without resistance or reporter genes) deletion of both ORFs that was corroborated by PCR.

To obtain the double mutant strain incapable of producing the cellulose and MLG polysaccharide in *R. etli*, the suicide vector pK18ΔbgsA was introduced into the Ret ΔcelAB genetic background by biparental conjugation with the *E. coli* S17.1 strain. After the allelic exchange, it was verified by PCR that the strain was a carrier of the two deletions.

### 2.4. Preparation of mRNA and Quantitative RT-PCR Assay

RNA extractions for real-time RT-PCR were performed using the Qiagen RNeasy RNA purification kit (Qiagen, Hilden, Germany) and following the manufacturer’s instructions. Total RNA (1 µg) treated with RNase-free Dnase I Set (Roche) was reverse-transcribed using random hexamers (Roche, Basel, Switzerland) as primers and Superscript III reverse transcriptase enzyme (Invitrogen). Quantitative real-time PCR was performed on an iCycler iQ5 (Bio-Rad, Hercules, CA, USA). Each 25 µL reaction contained 1 µL cDNA, 200 nM of each primer, and iQ SyBrGreen Supermix (Bio-Rad, Hercules, CA, USA). Control PCRs of the RNA samples were also carried out to confirm the absence of genomic DNA contamination. Samples were initially denatured by heating at 95 °C for 3 min, followed by a 35-cycle amplification and quantification program (95 °C for 30 s, 55 °C for 45 s and 72 °C for 45 s). A melting curve analysis was performed to ensure amplification of a single product. The efficiency of each primer pair (*E*) was determined with *R. etli* genomic DNA as template by running 10-fold serial dilutions (four dilution series) of and generating a standard curve by plotting the log of the dilution factor against the *C*_T_ value during amplification of each dilution. Amplification efficiency (*E*) was calculated using the formula [*E* = (10^(1/*a*)^ − 1) × 100], where *a* is the slope of the standard curve. The relative expression of the *bgsB* (*RHE_PE00362*) and *bgsA* (*RHE_PE00363*) genes was normalized to that of 16S rRNA, which was used as reference gene.

### 2.5. Calcofluor Binding Assays

Quantification of the CF were performed as follows: starting cultures of the different strains of *R. etli* strains were prepared as detailed above. Similar growth of all strains was confirmed. Culture were washed with MM and diluted 1/100 into 10 mL flasks containing MM supplemented with CF (100 μM final concentration) at 28 °C under agitation for 48 h. Cultures of the different strains were then centrifuged for 10 min at 4000 rpm. Supernatant containing broth with unbound CF was removed and the pellet was suspended in 2 mL of distilled water and disposed in wells of 24-well plates. CF binding measurements for three biological replicates of each strain were performed in a PTI fluorimeter (Photon Technology International, Birmingham, NJ, USA), expressing the results in arbitrary units ± standard error.

### 2.6. Biofilm Assays

Starting cultures of the different strains of *R. etli* strains were prepared as detailed above. Similar growth of all strains was confirmed. Culture were washed with MM and diluted up to OD_600_ = 0.1 in sterile MM. 200μL samples of diluted strains were aliquoted into the wells of sterile 96-well polystyrene plates (Sarstedt) and were incubated in a humid environment at 28 °C for 72 h. Afterwards, the liquid from the wells was removed and wells were washed with 240 μL of deionized water. A total of 240 μL of crystal violet (0.1% in water) was added to each well and left to stain for 1 h. The crystal violet was removed and each well was washed carefully with 3 × 240 μL of deionized water. A total of 240 μL of 70% ethanol was added to each well and the plate was gently agitated for at least 1 h. Appropriate dilutions were quantified by measurement of A_550_ in a microplate reader (Sunrise Tecan, Hombrechtikon, Switzerland). 

### 2.7. β-Glucan Isolation and Characterization

MLG from *R. etli* was purified as previously described for *S. meliloti* [7]. Starting cultures of *R. etli* Tn7pleD*Km were prepared in TY as indicated above. The starting culture was diluted 1/100 in 5 liter flasks containing 0.5 liters of liquid MM supplemented with Tc and grown with slow shaking (80 rpm) for 3 days at 28 °C. The flocs generated were recovered using a sieve of 500 µm cut-off. The flocs recovered were disposed in a 50 mL falcon tube and washed with 30 mL of MQ boiling water. After 5 min of boiling, the material was chilled out at room temperature and centrifuged for 20 min at 4000 rpm. The supernatant was discarded and the washing process repeated 4 times. The β-Glucan obtained was finally frozen and lyophilized overnight. Five milligrams of polysaccharides were digested with 10 units of a commercial lichenase (endo-1,3 (4)-β-glucanase; Megazyme) in 1 mL of water overnight at room temperature, including respective controls without the enzyme. Samples were centrifuged at 16,000× *g* for 10 min and then supernatants were frozen and lyophilized for storage. Samples were dissolved in water and studied by TLC on silica gel 60 Alugram SilG/UV (Macherey-Nagel, Düren, Germany) using butanol:acetic acid:water (2:1:1) as eluent. Carbohydrates were detected with orcinol:sulfuric acid [25]. Glucose and a mixture oligosaccharides were used as size markers: maltose, maltotriose, maltotetraose, maltopentaose, and maltohexaose (Sigma-Aldrich, St. Louis, MO, USA).

### 2.8. Plant Root Binding Assays

Bean seeds (*Phaseolus vulgaris* cv. Negro Jamapa) were surface-sterilized by treatment (2 × 5 min) with ethanol 100%. Ethanol was removed by washing three times with deionized water and a second treatment was carried out by adding sodium hypochlorite 5% for 5 min. Seeds were washed with abundant sterile deionized water to remove any remains of sodium hypochlorite. Bean seeds were germinated in purified agar:water 1% for 72 h at 28 °C in the dark. 

Seedlings were placed in 50 mL urine cups in which three holes were made in the lid to be able to introduce the root of the seedlings, so that the root was immersed in the nutrient solution and the cotyledon remained on the surface of the cap. The seedlings were incubated for 48 h in the dark. Next, taking only the lid, the seedlings were transferred to another urine cup containing 50 mL of the bacterial mixture prepared in nutrient solution. The cups were kept under gentle agitation (120 rpm) for 12, 24 or 48 h. After this time, roots were separated from the shoots and 3 groups of 3 roots each were separately taken and washed vigorously four times with 10 mL of sterile deionized water in Falcon tubes, to finally add 10 mL of MM with 2 mM EDTA. In order to be able to differentiate the two strains in competition, when it was possible the serial dilutions were seeded in plates of MM medium added with CR. When selection with CR was not possible, the strains to compete were selected for carrying different resistance to antibiotics. Finally, the percentage of each strain adhered to the root was determined.

### 2.9. Symbiotic Assays

Bean (*Phaseolus vulgaris* cv. Negro Jamapa) seeds were surface-sterilized and germinated as above. To test the infectivity of *R. etli* strains, 12 bean seedlings were sown in Leonard-type assemblies containing a mix vermiculite:perlite (3:1) on the top part and nitrogen-free nutrient solution [26] at the bottom. Each seedling was inoculated with 10^6^ CFU of the different *R. etli* strains. Bean plants were cultivated in a growth chamber with 16/8-h light/dark photoperiod at 24/16 °C day/night and 75% relative humidity. 

The shoot fresh weight and the number and fresh weight of nodules were determined after 29 days. Shoot and nodules dry weights were determined after desiccating the samples in an oven (65 °C) for 3 days. Dry shoots were ground and total nitrogen contents were determined following the Dumas method at the Ionomics Service of the institute CEBAS-CSIC (Murcia, Spain; http://www.cebas.csic.es/general_english/ionomics.html (accessed on 20 August 2022)). 

### 2.10. Nodules Histology and Microscopy 

Nodules induced on *Phaseolus vulgaris* by *R. etli* were fixed and embedded according to a slightly modified protocol of Redondo et al. [27]. Freshly collected nodules were immediately fixed under vacuum for 2 h at 4 °C in 5% glutaraldehyde and 4% paraformaldehyde in 100 mM Na-cacodylate buffer (pH 7.4) containing 25 mg/mL Sucrose. After a second fixation for 1.5 h at 4 °C, three washes of 1 h with Sucrose-cacodylate buffer were carried out. Then nodules were postfixed in 1% osmium tetroxide in the same buffer (16 h at 4 °C). Samples were dehydrated in ethanol series, including an incubation in 1% uranile in 70% ethanol for 24 h at 4 °C. Samples were embedded in Unicryl resin and polymerized for 24 h at 60 °C.

Semithin (1 μm) sections of nodules were sliced with a Reichert Ultracut S ultramicrotome fitted with a diamond knife. Sections for light microscopy were stained with 1% (*w*/*v*) toluidine blue in aqueous 1% sodium borate for direct observation with a Zeiss Axioskope photomicroscope. 

### 2.11. Phylogenetic Analyses

Protein sequence similarity searches were carried out with the BLASTP program from NCBI [28]. Alignments were performed with CLUSTALW [29]. Phylogenetic and molecular evolutionary analyses were conducted using MEGA, version 11 [30]. The following phylogenetic parameters were used: Analysis, Phylogeny Reconstruction; Statistical Method, Neighbor-Joining; Number of Bootstrap Replications, 500; Substitutions Type, amino acid; Model/Method, Poisson model; Rates among Sites, Uniform rates; Pattern among Lineages, Same (Homogeneous); Gaps/Missing Data Treatment, and Pairwise deletion.

### 2.12. Statistical Analyses

Statistical analyses were made with GraphPad Prism 6.01 for Windows (GraphPad Software, New York, NY, USA) following an unpaired, two tailed, homocedastic Student’s *t*-test. 

## 3. Results

### 3.1. Rhizobium etli CFN42 Produces Two Linear β-Glucans Activated by c-di-GMP

Three different linear β-glucans have been described so far in different members of Rhizobiaceae family: curdlan, cellulose, and the recently discovered Mixed-Linkage β-glucan or MLG. Their biosynthetic operons include genes coding for at least one GT involved in the formation of glycosidic bonds by the addition of the UDP-Glucose to the nascent polysaccharide [31]. According to the carbohydrate-active enzyme database (CAZY), the *R. etli* CFN42 genome (Ret) encodes 71 hypothetical GTs ([32]; www.cazy.org (accessed on 20 August 2022)). A Clustal W alignment and a subsequent phylogenetic analysis were performed with these 71 Ret GTs, plus three reported GTs involved in the synthesis of these linear β-glucans from different Rhizobiaceae strains: Cellulose synthase (CelA; Atu3309), Curdlan synthase (CrdS; Atu3056) from *A. tumefaciens* C58 and MLG synthase (BgsA; SM_b20391) from *S. meliloti* 8530 (Sme). The Neighbor-Joining tree shows (99% of bootstrap value) that two of these GTs: Q2K9Z1 (RHE_CH01542) and Q2K103 (RHE_PE00363), are close related to the cellulose (CelA; Atu3309) and MLG synthases (BgsA; SM_b20391), respectively (Figure S1). RHE_CH01542 is included, with other 3 *cel/bcs* genes, in a typical cellulose operon located in the chromosome (Figure S1). Indeed, RHE_CH01542 has been reported as the cellulose synthase of Ret CFN42 strain [19]. RHE_PE00363 is located in a putative bicistronic operon in the plasmid p42e of the strain. It is preceded by RHE_PE00362, which displays 67% of identity with BgsB of Sme and also contains a HlyD-like domain likely involved in the formation of a channel to expel MLG out of the cell (Figure S1). In contrast, among the 71 hypothetical GTs of Ret, the Neighbor-Joining tree does not show a clear homologue to the curdlan synthase of *A. tumefaciens* (CrdS; Atu3056; Figure S1). Furthermore, a Blast X using the DNA sequence coding for the full operon (Atu3055-Atu3057) as a query against the Ret genome did not show the presence of this *crd* tricistronic operon (*crdA-crdC-crdS*) in CFN42 strain, suggesting that Ret CFN42 does not contain genes for curdlan production.

**Figure 1:**
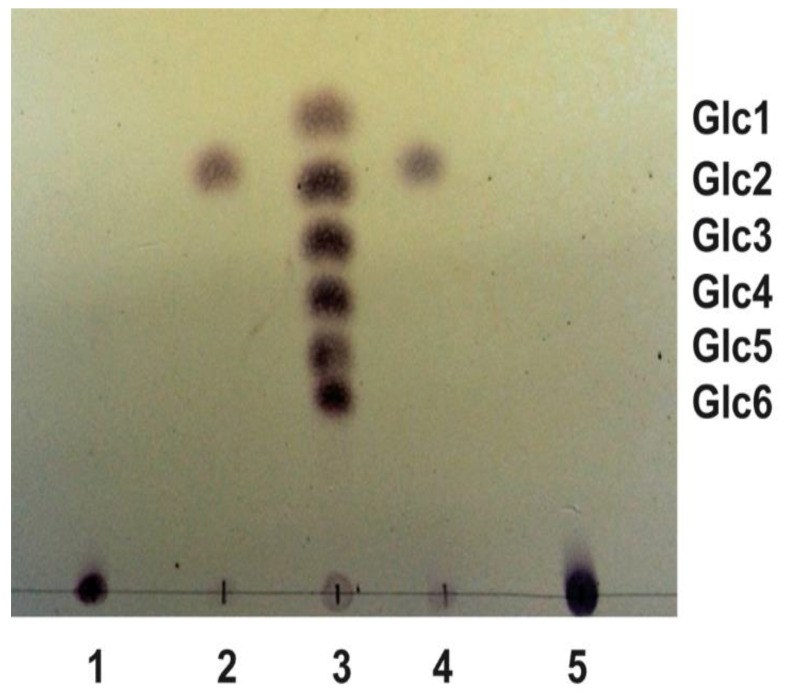
TLC of β-glucans hydrolyzed with lichenase. Lane 1: untreated *R. etli* ML β-Glucan; lane 2: *R. etli* ML β-Glucan incubated with lichenase; lane 3: mixture of glucose, maltose, maltotriose, maltotetraose, maltopentaose, and maltohexaose; lane 4: *S. meliloti* ML β-Glucan incubated with lichenase; lane 5: untreated *S. meliloti* ML β-Glucan.

Rising the intracellular levels of c-di-GMP by introducing the pleD* gene from *Caulobacter vibrioides* (formerly *Caulobacter crescentus*) into Ret generated wrinkled colonies strongly stained in media with Congo Red (CR^+^) or Calcofluor (CF^+^) [19]. This wrinkled phenotype is, at least in part, generated by the production of cellulose, activated under high c-di-GMP conditions, because a Ret Cel^−^ mutant (RetΔcelAB) expressing PleD* showed a reduced CR and CF staining in comparison with the wild-type [19]. However, a Ret Cel^−^ mutant still significantly bound these dyes, i.e., CF-derived fluorescence was approximately 50% of the wild-type, suggesting the presence of another c-di-GMP activated EPS [19]. MLG has been reported as another β-glucan activated by c-di-GMP giving CR^+^ and CF^+^ phenotypes in *S. meliloti* 8530 [7]. Since Ret contains a *bgsBA*-like operon, we wondered if Ret is able to produce MLG in addition to cellulose under high c-di-GMP conditions. A protocol for the purification of MLG was followed with a Ret Cel^−^ mutant expressing PleD*. A parallel purification experiment with Sme pJBpleD* was carried out as a positive control. Once purified, both samples were subjected to digestion with lichenase and the product of the hydrolysis separated by TLC. Sme MLG can be hydrolyzed specifically by lichenase, an endohydrolase that cleavages all β (1→4) bonds, immediately following β (1→3) bonds, generating a disaccharide [D-Glu-(1→3)-β-D-Glu] as the only product of hydrolysis [7]. After lichenase digestion and the TLC separation, the sample from Ret generated a disaccharide indistinguishable from the Sme MLG digestion (Figure 1). This disaccharide is specific from the lichenase digestion as it was not observed in the absence of the enzyme (Figure 1). This result supports that, under high c-di-GMP conditions, Ret is able to produce a MLG that is structurally identical to Sme MLG.

### 3.2. Cyclic-di-GMP Activation of MLG Production by Rhizobium etli CFN42

In Sme, MLG production is activated upon binding of c-di-GMP to the C-terminus of BgsA [17], whereas *bgsBA* gene transcription is dependent on the ExpR/SinI quorum sensing system [7]. Although Ret has several quorum sensing systems, a transcriptional regulator equivalent to ExpR of Sme has not been described [33]. Hence a different MLG regulation is plausible in Ret. In order to evaluate if the positive effect over MLG production exerted by c-di-GMP occurs at transcriptional level, expression of Ret *bgsB* and *bgsA* genes (RHE_PE00362 and RHE_PE00363, respectively) were analyzed in the presence or absence of pleD* by RT-PCR experiments. Cel^−^ mutant strains of Ret were used in order to avoid the high flocculation promoted by cellulose in the presence of pleD* and to facilitate handling the samples during RNA extraction. The presence of pleD* did not produce an increment of the expression of neither *bgsB* or *bgsA* genes (Figure S2A). Conversely, MLG biosynthetic genes seem to be slightly repressed (1.76 y 2.35 times for *bgsB* y *bgsA*, respectively) under high c-di-GMP conditions (Figure S2A). This result indicates that similar to Sme, c-di-GMP does not regulate MLG production at transcriptional level in Ret. 

An alignment of the C-terminal cytoplasmic domain of BgsA_Ret_ and BgsA_Sme_ shows that all residues required for both c-di-GMP binding and MLG activation in BgsA_Sme_ are fully conserved in BgsA_Ret_ (Figure S2B). This suggests that similar to Sme, c-di-GMP is an allosteric activator of MLG also in Ret, through its binding to the C-terminal cytoplasmic domain of BgsA.

### 3.3. Cyclic-di-GMP Activation of Cellulose and MLG Production in Rhizobium etli CFN42

In order to evaluate the impact of c-di-GMP on the production of these two β-glucans in Ret, mutants unable to produce cellulose (Cel^–^ MLG^+^), MLG (Cel^+^ MLG^–^) or both β-glucans (Cel^–^ MLG^–^) were constructed (Table 1). In addition to the previously reported Cel^−^ mutant (RetΔcelAB; [19]), an in-frame deletion mutant in the MLG GT gene (*bgsA*, RHE_PE00363) was generated. The RHE_PE00363 deletion was also introduced into a Cel^−^ mutant background (RetΔcelAB), thereby obtaining a double mutant impaired in the production of both β-glucans (Cel^–^ MLG^–^). Later, a mini-Tn7 transposon containing the pleD* gene (Tn7pleD*Km) was introduced into each wild-type and mutant genomes. As controls, isogenic strains were constructed after insertion of a mini-Tn7 carrying no pleD* (Tn7Km). Since Tn7 inserts into a specific site of the genome, the precise insertion sites were verified by sequencing (Table 1).

**Table 1:** Bacterial strains and plasmids used in this study.

Strains	Relevant Characteristic	Reference or Source
Rhizobia strains		
*R. etli* CFN42	Ret; Wild-type	[34]
*S. meliloti* pJBpleD*	Sme 8530 strain with pJBpleD* plasmid	[7]
Ret Tn7Km	Ret with a mini-Tn7Km transposon	[23]
Ret Tn7pleD*Km	Ret with a mini-Tn7pleD*Km transposon	[23]
Ret Tn7pleD*Tc	Ret with a mini-Tn7pleD*Tc transposon	[23]
LR102	Ret Cel^−^ Tn7Km; CFN42 Δ*celAB* with a mini-Tn7Km transposon	This work
LR101	Ret Cel^−^ Tn7pleD*Km; CFN42 Δ*celAB* with a mini-Tn7pleD*Km transposon	This work
LR104	Ret MLG^−^ Tn7Km; CFN42 *ΔbgsA* (*RHE_PE00363*) with a mini-Tn7Km transposon	This work
LR103	Ret MLG^−^ Tn7 pleD*Km; CFN42 *ΔbgsA* (*RHE_PE00363*) with a mini-Tn7pleD*Km transposon	This work
LR106	Ret Cel^−^ MLG^−^ Tn7Km; CFN42 Δ*celAB* and Δ*bgsA* with a mini-Tn7Km transposon	This work
LR105	Ret Cel^−^ MLG^−^ Tn7pleD*Km; CFN42 Δ*celAB* and Δ*bgsA* with a mini-Tn7pleD*Km transposon	This work
*Escherichia coli* strains		
DH5α	*supE44*, ∆*lacU169*, Φ80, *lacZ*∆M1, *recA1*, *endA1*, *gyrA96*, *thi1*, *relA1*, *5hsdR171*	[35]
S17.1	Tmp^r^, Sm^r^, Sp^r^; *thi*, *pro*, *recA*, *hsdR*, *hsdM*, Rp4Tc::Mu, Km::Tn7	[36]
β2163	(F−) RP4-2-Tc::Mu *_dapA*::(*erm-pir*) [Km^r^ Em^r^]	[22]
OmniMAX	*F′* [*pro*AB*+ lacIq lac*ZΔM15 *Tn10(TetR)* Δ*(ccdAB)*] *mcr*A Δ*(mrr-hsdRMS-mcr*BC*) φ*80*(lacZ)*ΔM15 Δ*(lacZ*YA*-argF)* U169 *end*A1 *rec*A1 *sup*E44 *thi-1 gyr*A96 *rel*A1 *ton*A *pan*D	Invitrogen
Plasmids		
pK18*mobsacB*	Km^r^; mobilizable suicide plasmid	[24]
pK18ΔbgsA	Km^r^; pK18*mobsacB* carrying the deleted version of the *bgsA*(*RHE_PE00363*) gene	This work
pCR-2.1-TOPO	Km^r^ Ap^r^; cloning vector for PCR products	Invitrogen
TOPOΔbgsA	Km^r^ Ap^r^; pCR-2.1-TOPO carrying the deleted version of *bgsA*(*RHE_PE00363*) gene	This work

Ap^r^, Km^r^, Sm^r^, Sp^r^, Tc^r^, Tmp^r^, Em^r^ stand for resistance to ampicillin, kanamycin, streptomycin, spectinomycin, tetracycline, trimethropin and erythromycin, respectively.

Mini-Tn7pleD*Km insertion into the *R. etli* genome leads to more than 2-logs increments of the intracellular levels of c-di-GMP, while control Tn7Km transposants show no changes in c-di-GMP levels nor any other differential trait [23]. 

**Figure 2:**
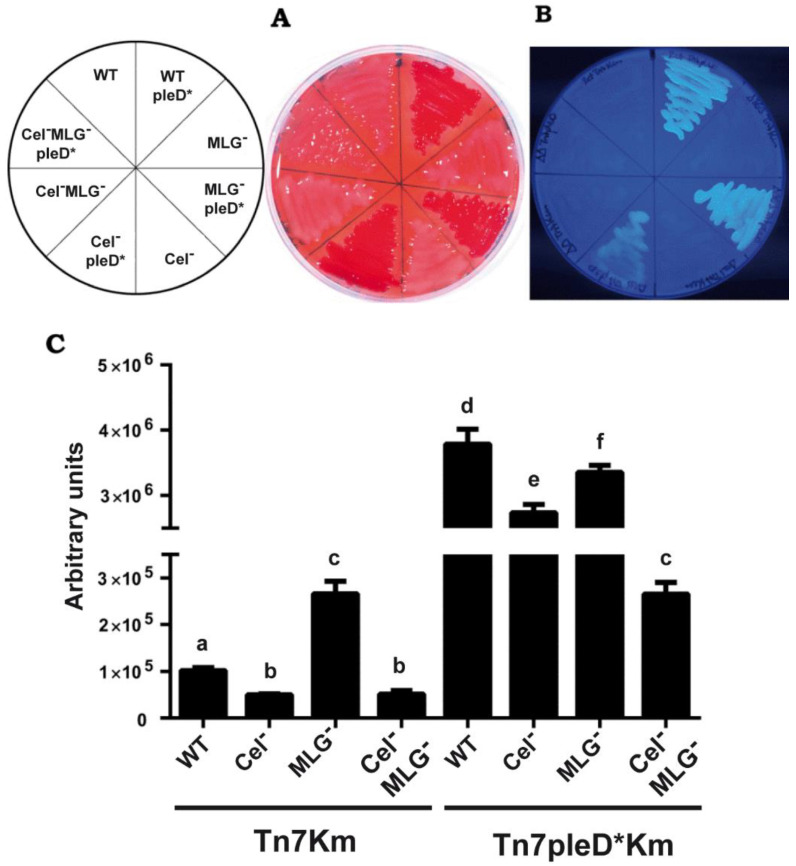
Contribution of cellulose and MLG to the CR^+^ and CF^+^ phenotypes of *R. etli* CFN42. Single mutants in *celAB* cellulose genes (Cel^–^), in *bgsA* (MLG^−^), the double mutant (Cel^–^ MLG^–^) and their respective parental strains (WT) with or without pleD*, were grown in minimal medium added with Congo Red ((**A**), 125 μg/mL) or Calcofluor ((**B**), 200 μg/mL). Plates were photographed after 48 h of growth at 28 °C. (**C**) Quantification of Calcofluor-derived fluorescence emitted by liquid cultures in minimal medium. Bars represent the mean of three biological replicates with three technical replicates ± standard deviation. Different letters over the bars indicate statistical significant differences with a *p* < 0.05.

Ret strains were grown on solid MM supplemented with CR or CF to evaluate cellulose and MLG production. As expected, the presence of pleD* in Ret promoted strong staining with both dyes, generating CR^+^ and CF^+^ colonies, which were not observed in the wild-type strain under physiological c-di-GMP conditions (Figure 2). This result suggests that the production of both β-glucans in the wild type Ret CFN42 is repressed under the conditions tested. Interestingly, single mutants impaired in either cellulose or MLG production, also stained with CR and CF in the presence of pleD* (Figure 2A,B). In contrast, the double mutant (Cel^–^ MLG^–^) with pleD* generated CR^–^ and CF^–^ colonies, indistinguishable from those in the absence of pleD*. This suggests that both β-glucans are activated by high intracellular levels of c-di-GMP and contribute together to the CR^+^ and CF^+^ phenotypes. The results also indicate that the production of at least one of these β-glucans, cellulose or MLG, is required for the CR^+^ and CF^+^ phenotypes (Figure 2A,B). These results were confirmed by the quantification of CF-derived fluorescence of cultures grown in liquid MM supplemented with CF (Figure 2C).

Ret overexpressing pleD* displayed 37 times more CF-derived fluorescence than the wild-type. This positive effect of c-di-GMP was slightly reduced in either single cellulose or MLG mutants (Figure 2C). Double mutants displayed a drastic reduction of CF-derived fluorescence, thus confirming that both cellulose and MLG are the main contributors to CF^+^ phenotype in Ret under high c-di-GMP conditions (Figure 2C). This corroborates the simultaneous production and contribution of these two β-glucans to the CF-derived fluorescence by Ret under high c-di-GMP conditions. However, CF-derived fluorescence of the double mutant (Cel^−^ MLG^−^) in the presence of pleD* were still slightly (2.5 times) higher than the wild-type under physiological c-di-GMP conditions, suggesting that c-di-GMP could be activating one or more additional EPS(s) in Ret. These could be the unipolar polysaccharide (UPP) described in *A. tumefaciens* [37], also present in Sme [15], or the recently discovered arabinose-containing polysaccharide (APS) [16], which have been reported to be activated by c-di-GMP and to contribute to CR^+^ phenotype in different members of Rhizobiaceae family. As discussed above, the Ret genome contains putative operons resembling those reported for UPP and APS biosynthesis (Figure S1).

### 3.4. Role of Cellulose and MLG in Rhizobium etli CFN42 Aggregation and Biofilm Formation

**Figure 3:**
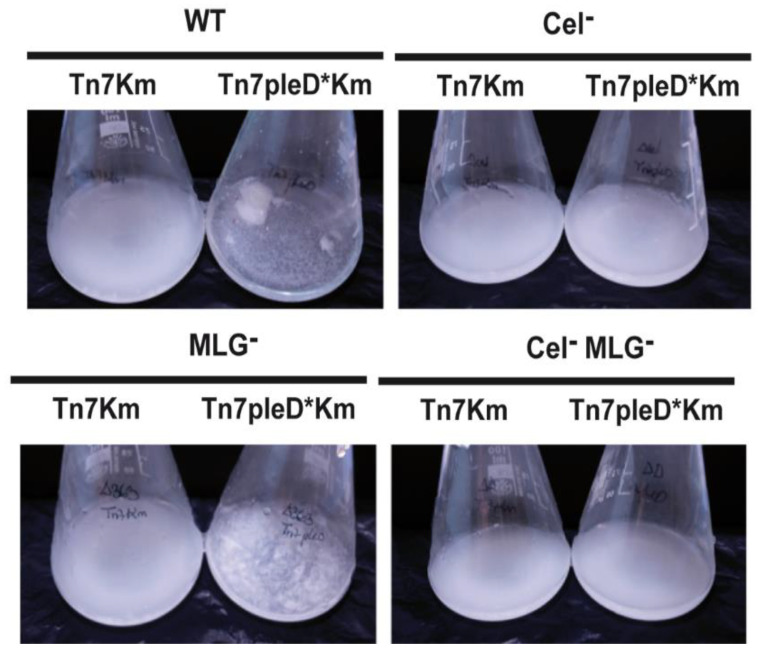
Role of the β-glucans Cellulose and MLG in the flocculation of *R. etli CFN42*. Single mutants in *celAB* cellulose genes (Cel^−^), in *bgsA* (MLG^−^), the double mutant (Cel^−^ MLG^−^) and their respective parental strains (WT) with or without pleD* were grown in minimal medium with shaking. Flasks were photographed after 24 h of growth at 28 °C to observe the flocs.

We evaluated the role of these two β-glucans in different Ret free-living phenotypes associated with an aggregative behavior. Single and double mutants unable to produce cellulose and/or MLG were assayed for flocculation and biofilm formation experiments under physiological and high c-di-GMP conditions. As previously reported [19], the overexpression of the pleD* gene in Ret wild-type strain induces a strong aggregative behavior with bacteria forming flocs after overnight culture in liquid media (Figure 3).

**Figure 4:**
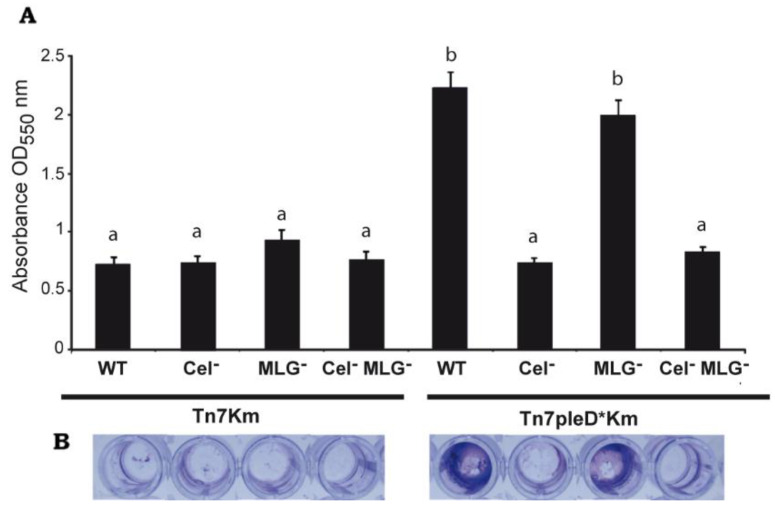
Role of Cellulose and MLG in biofilm formation by *R. etli CFN42*. (**A**) Biofilm formation by single mutants in *celAB* cellulose genes (Cel^−^), in *bgsA* (MLG^−^), the double mutant (Cel^−^ MLG^−^) and their respective parental strains (WT) with or without pleD* quantified after static growth, 72 h in MM in a 96-well plate at 28 °C, by crystal violet (CV) staining and represented as the means of eight different wells for each strain ± standard deviation of three independent experiments. Similar growth of all strains was confirmed by measuring OD_600_ before CV staining. (**B**) Representative pictures of biofilms adhered to the wells of the polystyrene plates, stained with CV, before resuspension with ethanol. Different letters over the bars indicate statistical significant differences with a *p* < 0.05.

Interestingly, the MLG^−^ mutant with pleD* (Ret ΔbgsA Tn*7*pleD*Km) was still able to produce flocs, although visually of smaller size (Figure 3). On the contrary, the single Cel^−^ and the double Cel^−^ MLG^−^ mutant with pleD* had lost their ability to flocculate in these conditions, showing a similar behavior than the strains with physiological levels of c-di-GMP (Figure 3). These results support that cellulose is the main β-glucan involved in Ret aggregation and flocculation. Next, we studied the impact of these two β-glucans on Ret biofilm formation. Under physiological c-di-GMP conditions, no significate differences were observed among the different cellulose and/or MLG producer strains (Figure 4). However, under high c-di-GMP conditions, the wild-type strain and the MLG^−^ single mutant were both able to form a dense biofilm quantifiable by crystal violet, indicating that MLG production is not required for Ret biofilm formation under these conditions (Figure 4).

In contrast, mutants impaired in cellulose production (single Cel^−^ and the double Cel^−^ MLG^−^ mutant) had lost their ability to form biofilm under high c-di-GMP conditions, showing a more similar level than the strains in the absence of pleD* (Figure 4). As for flocculation, these experiments indicate that cellulose is the main β-glucan involved in the biofilm formation by Ret under the conditions tested.

### 3.5. Role of Cellulose and MLG in Rhizobium etli CFN42 Adhesion to Plant Roots

**Figure 5:**
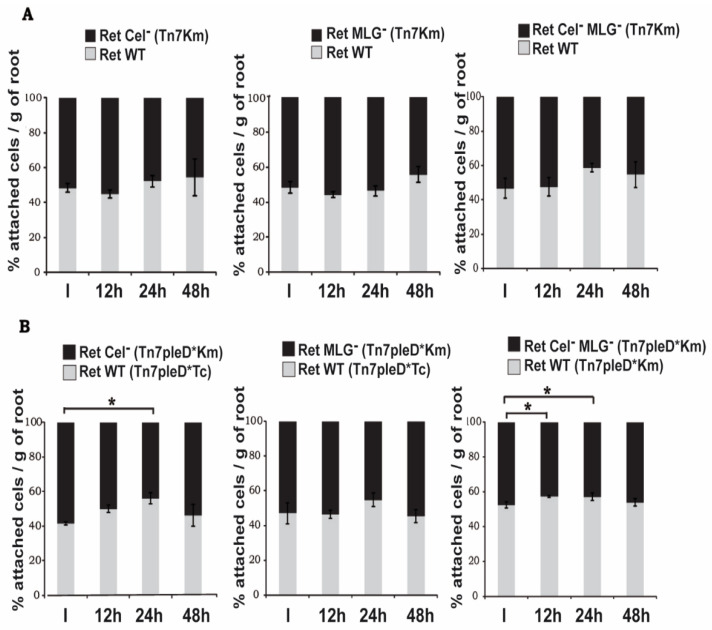
Role of β-glucans Cellulose and MLG in the adhesion of *R. etli* CFN42 to bean roots. 1:1 mixture (inoculum; I) of the different strains were in contact with bean roots for 12, 24, or 48 h. (**A**) Competitive adhesion under physiological conditions of c-di-GMP. (**B**) Competitive adhesion under of high levels of c-di-GMP conditions (presence of pleD*). The bars indicate the proportion of CFU of the mutants (in black) and of the control strains (in grey) ± SEM. Asterisks indicate significant differences using an analysis of variance with a level of significance α= 0.1.

We have previously reported that increasing c-di-GMP levels in Ret would favor the early stages of the symbiotic interaction due to enhanced adhesion to plant roots [19]. Thus, we wanted to evaluate whether these two c-di-GMP-regulated β-glucans may have a function in bacterial adhesion to host roots. We studied the impact of these two β-glucans on Ret attachment to the roots of *Phaseolus vulgaris* in competitive adhesion experiments, both under high and physiological c-di-GMP levels. In order to facilitate their identification, different mutant strains were chromosomally labelled with mini-Tn7 transposons harboring different antibiotic markers (Tc or Km). For competitive adhesion experiments under physiological c-di-GMP levels, the wild-type strain was tested against the different β-glucan mutants tagged with a mini-Tn*7*Km transposon (ratio 1:1). No significant differences were observed between the wild-type and any of the β-glucan mutants tested (Figure 5A). The competitive adhesion experiments were carried out with the plant growing in nutritive solution under hydroponic conditions. Our results suggest these β-glucans are not significantly produced by Ret under the conditions tested. Thus, we also carried out the competitive adhesion experiments between the wild type and the β-glucans mutants expressing pleD*, therefore with the production of both β-glucans activated. When possible, i.e., Ret wild-type strain (Ret Tn7pleD*Km) versus Cel^−^ MLG^−^ double mutant (Ret Cel^−^ MLG^−^ Tn7pleD*Km), all carried the same antibiotic resistant marker and plate counting was done in media supplemented with CR, which allowed for clearly discriminating between both colony types (CR^+^ vs. CR^−^). Single mutants in cellulose (Cel^−^ MLG^+^) and MLG (Cel^+^ MLG^−^) were labelled with Tn7pleD*Km and tested in competition with a wild-type labelled with Tn7pleD*Tc; in these cases, the different antibiotic resistances allowed to differentiate both competitors and facilitated plate counting. Under high c-di-GMP conditions, the wild-type outcompeted the mutants impaired in cellulose production (either single Cel^−^ or the double Cel^−^ MLG^−^ mutant), with the highest differences 24 h after inoculation (Figure 5B). However, these slight, albeit statistically significant differences, disappeared after longer incubation times, with cellulose mutants showing similar attachment rates than the wild-type at 48 h. On the contrary, no significant differences were observed between the wild-type and the single MLG^−^ mutant (Figure 5B). These experiments suggest that under elevated c-di-GMP conditions, cellulose but not MLG contributes to the adhesion of Ret to the roots of its host plant. Anyway, the importance of both β-glucans in the attachment of Ret to the host plant roots seem to be limited under the condition tested, since a double mutant impaired in the production of both β-glucans (Cel^−^ MLG^−^) still bound to roots at high rates (Figure 5B).

### 3.6. Role of Cellulose and MLG in Rhizobium etli CFN42 Symbiotic Interaction with the Legume Plant

**Figure 6:**
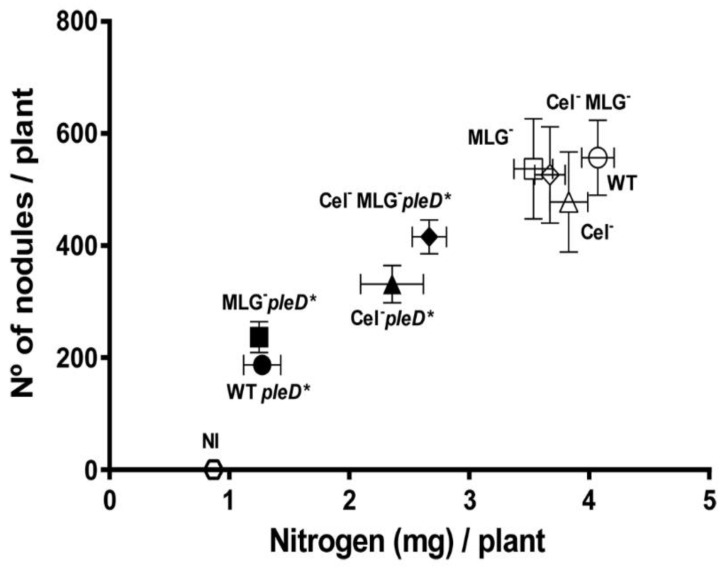
Role of β-glucans cellulose and MLG in the symbiotic interaction of *R. etli* CFN42 with bean plants. The symbols indicate the mean ± SEM of the nitrogen content of shoot versus the number of nodules per plant inoculated with single mutants in *celAB* cellulose genes (Cel^−^), in *bgsA* (MLG^−^), the double mutant (Cel^−^ MLG^−^) and their respective parental strains (WT) with (in black) or without pleD* (in white) plus the non-inoculated (NI) after 29 days.

Under physiological c-di-GMP levels, cellulose and MLG mutants did not show significant differences regarding the wild-type during early stages of the interaction with the host, e.g., attachment to the plant roots (Figure 5). We wanted to know if these two β-glucans would have an important role in later stages of the interaction with the legume plant. Thus, single and double mutants in cellulose and/or MLG production were assayed, together with the wild-type, in their symbiotic association with beans grown in Leonard-type assemblies. Different symbiotic and plant growth related parameters were determined, including (i) shoot fresh and dry weight, (ii) shoot nitrogen content, and (iii) the number and the fresh and dry weight of the nodules formed 4 weeks post inoculation. Under physiological levels of c-di-GMP, wild-type and β-glucans mutants formed effective, nitrogen-fixing symbioses with *P. vulgaris*. No significant differences were observed among the different mutants and wild-type in any of the different plant biomass parameters measured, showing a weight average (mean ± SEM) of 9.16 ± 1.18 and 1.29 ± 0.17 of fresh and dry shoot, respectively, while not inoculated plants showed 1.80 ± 0.18 and 0.33 ± 0.05 (Figure S3). No significant differences were observed in the number or weight of the nodules formed, with an average of 525 ± 83 nodules per plant and a fresh and dry weight of 1.66 ± 0.25 and 0.32 ± 0.05, respectively (Figure S3). However, the shoot nitrogen content was slightly, albeit statistically significant, reduced in plants inoculated with the single MLG mutant (MLG^−^; *p* = 0.02) and the Cel^−^ MLG^−^ double mutant (*p* = 0.04), containing 86% and 90%, respectively, regarding wild-type (Figure S3). Furthermore, a representation of the nitrogen content of shoot versus the number of nodules per plant showed that both β-glucans mutants seem to be slightly affected in their symbiotic interaction with the host (Figure 6). Altogether, the data indicate that under physiological c-di-GMP conditions, the ability to produce cellulose and/or MLG is not essential to establish effective symbiosis, albeit MLG could help to achieve highest efficiency levels. 

The presence of high c-di-GMP levels has a strong negative impact on late stages of Ret symbiotic interaction with its legume host [19]. As shown above, under this high intracellular c-di-GMP conditions the production of both β-glucans is exacerbated (Figure 2). In order to test if the continued overproduction of cellulose and/or MLG along the whole nodulation and infection process could be responsible for the negative effects observed, we compared the symbiosis efficiency of the wild-type and β-glucans mutants, expressing high levels of intracellular c-di-GMP. Compared to physiological c-di-GMP, all wild-type and mutants expressing PleD* formed symbioses with reduced nitrogen fixation efficiency. All parameters measured, aerial biomass accumulation and N contents, number of nodules, and biomass were reduced in the PleD* strains (Figure S3). These results are in agreement with previous studies, indicating that the high c-di-GMP produced by PleD* has a pernicious effect over the nitrogen fixation [19]. Interestingly, the strongest reductions in all parameter were observed in the wild-type and the MLG^−^ single mutant under high c-di-GMP (Ret Tn7Km pleD*; Figure S3). This further indicated that the MLG overproduction was not the main reason for the symbiotic shortcoming provoked by high c-di-GMP (Figure 6 and Figure S3).

In contrast, a mutation in the cellulose GT, partially counteracted the negative effects of pleD*. Under these conditions, the single Cel^−^ mutant formed a more effective symbiosis than the wild-type (Figure S3). This suggests that overproduction of cellulose induced by c-di-GMP is detrimental for symbiosis establishment and nitrogen fixing efficiency. This partial improvement was even more pronounced in the double mutant Cel^−^ MLG^−^ (Figure S3). Indeed, according to their symbiotic capacity in the presence of pleD*, β-glucans mutants can be differentiated in two well separated groups (Figure 6). These results indicate that the overproduction of cellulose, and even more the overproduction of both β-glucans promoted by pleD*, have a harmful effect on the symbiotic association of Ret with the host plant. Nonetheless, compared to physiological c-di-GMP levels the presence of pleD* in the double mutant Cel^−^ MLG^−^ still generated a reduction of 21% (from 526 ± 86 to 416 ± 30) and 27% (from 3.67 ± 0.13 to 2,67 ± 0.14) of number of nodules and the nitrogen per plant, respectively (Figure 6 and Figure S3). This reduced symbiosis efficiency of the double mutant (Cel^−^ MLG^−^) clearly indicates that the high intracellular levels of c-di-GMP generated by pleD* do affect other cellular processes important for symbiosis but unrelated to the production of cellulose and MLG β-glucans. 

### 3.7. Impact of High c-di-GMP on Nodule Structure 

The high c-di-GMP contents induced by the expression of the DGC PleD* in *R. etli* are very detrimental for nodulation and symbiotic nitrogen fixation, which determines lower biomass production and reduced N contents of the host plant. In an attempt to understand the negative effects of high c-di-GMP contents in the symbiosis, we carried out a comparative histological study of the nodules formed by strains expressing PleD*. Nodules formed 2 and 3 weeks after inoculation were studied. Nodule sections were stained with toluidine blue and observed under the optical microscope. All nodules showed a normal appearance, with a central infection zone containing both infected and uninfected cells (Figure S4). We did not find clear differences between nodules formed by the wild-type strain and those formed by the same strain expressing PleD*. All nodules displayed similar morphology and internal organization, regardless of whether they were produced by the wild type or β-glucan mutants.

## 4. Discussion

Exopolysaccharides are synthesized by biomachinery complexes anchored to the membrane, which are strictly regulated in accordance with environmental and physiological cues in order to optimize the high energy and nutrient costs that EPS biosynthesis and secretion require. Another generalized feature is that EPS often share common regulatory elements. For instance, to date more than a dozen different EPS are known to be regulated by the bacterial second messenger c-di-GMP [12]. 

Bacteria have the ability to produce different β-glucans. Among the unbranched linear are the biotechnological relevant curdlan and cellulose, with β (1→3) and β (1→4) glycosidic linkages, respectively, and the recently discovered MLG with a perfect alternation of β (1→3) and β (1→4) glycosidic bonds. Interestingly, besides their related chemical structure, the three seem to be activated in bacteria by the second messenger c-di-GMP, albeit likely through different mechanisms. Cellulose is by far the best known and cyclic-di-GMP can activate its production at transcriptional and post-translational levels. This second messenger stimulates the expression of the transcription factor CsgD [38,39], which in turn activates the expression of the cellulose biosynthetic genes in enterobacteria [40,41,42]. Cyclic-di-GMP is also an allosteric activator, particularly through its binding to the PilZ domain of cellulose synthase CelA protein [43,44]. The regulation is further complicated by the fact that CsgD may also regulate DGCs that impact on the c-di-GMP levels required by cellulose synthase activation in *E. coli* [45,46]. An indirect regulation of curdlan production at transcriptional levels by c-di-GMP has also been reported, but activation mechanism of curdlan synthesis is still unknown [47]. On the other hand, we recently reported the post-translational regulation of MLG through the specific binding of c-di-GMP to a novel domain located on the C-terminus of the MLG synthase BgsA [17], being the expression of the MLG biosynthetic genes (*bgsBA*) regulated by ExpR, a transcriptional regulator which operates in a quorum sensing system [7]. Thus, besides β-glucan GTs seems to share a common ancestor and all contain a conserved central catalytic CESA_CelA_like domain (cd06421) [7], they likely have greatly diverged in their regulatory schemes. The same second messenger can regulate the production of different β-glucans by targeting different effectors at multiple levels, a phenomenon that has been termed ‘sustained sensing’ and provides an additional level of complexity for the fine-tuned regulation that highly energy demanding polymers, such as β-glucans, require [48]. This broad variety of c-di-GMP regulation network is even more justified in bacteria, such as *R. etli*, that has the ability to produce more than one of these β-glucans at the same time. 

Our results indicate that significant cellulose and MLG production does not occur in wild type *R. etli* under free-living laboratory conditions. In *R. leguminosarum* bv. Trifolii, cellulose production was also shown to be absent in free-living cultures and was suggested to be induced upon contact with plant roots [49]. However, in other rhizobial strains significant cellulose production was shown to be constitutive [50]. Regarding MLG, *bgsBA* gene expression occurs in free-living cultures of *S. meliloti* in an ExpR/SinI dependent manner, however in these conditions MLG production is not observed unless there is a rising of intracellular c-di-GMP levels [7]. Likewise, *bgsBA* gene expression is observed in free-living *R. etli* but MLG production is only significant after artificial elevation of c-di-GMP, and *bgsBA* gene expression is not significantly affected by c-di-GMP in *R. etli*, which is also similar to *S. meliloti*. Considering the high sequence conservation between BgsA from *R. etli* and *S. meliloti*, it is likely that c-di-GMP activation of MLG production in *R. etli* occurs after binding of the dinucleotide to the C-terminal domain of BgsA. 

The production of both cellulose and MLG β-glucans in *R. etli* was triggered by increasing the intracellular levels of c-di-GMP upon expression of a heterologous DGC such as PleD*. Under an activated-c-di-GMP state both β-glucans contribute to the CF^+^ and CR^+^ phenotypes. However, *R. etli* seems to produce more cellulose than MLG in the presence of pleD*. It is possible that the indiscriminate rise of c-di-GMP caused by pleD*, is favoring the production of cellulose over MLG. This is directly connected with the eternal discussion about the “local” versus “general” pool of c-di-GMP in bacteria [46]. Nevertheless, it is also possible that the cellulose synthase competes better than the MLG synthase for UDP-glucose available in the cell, under this state of high production rates. Anyway, the fact that the sum of the CF-derived fluorescence values of single mutants were much higher than the wild-type strain, suggest that the loss of one of these β-glucans favors the production of the remaining, at least under this high c-di-GMP conditions. 

Cellulose and MLG do not equally contribute to the various bacterial phenotypes analyzed. Under our experimental conditions, cellulose appears to have a stronger impact than MLG on flocculation and biofilm formation. Cellulose also seemed slightly more relevant than MLG for competitive adhesion to plant roots. In contrast, MLG participates in bacterial aggregation and biofilm formation in the non-cellulose producer, such as *S. meliloti*, where it is also relevant for efficient attachment to the roots of its host plant [7]. It is possible that MLG may substitute the biological role of cellulose in *S. meliloti*, having then a marginal role in cellulose producing bacteria, similar to *R. etli*. However, it should be born in mind that in our experiments both cellulose and MLG have been forced to be produced by an artificial rising of c-di-GMP. It is plausible that in the wild-type situation with physiological c-di-GMP levels, MLG and cellulose could be subject to separate regulation and be produced under differential, yet unknown conditions. Thus, it is possible that MLG has a more relevant biological role in *R. etli*, as discussed below. 

Some of the results of this work suggest that other c-di-GMP-regulated EPS might be operating in *R. etli*. The CF-derived fluorescence of the double mutant (Cel^−^ MLG^−^) in the presence of pleD* was 2.5-fold higher than the wild-type under physiological c-di-GMP conditions, suggesting that this second messenger might be activating an additional EPS in Ret. Indeed, biosynthetic clusters for APS and UPP were identified in *R. etli* (Figure S1). UPP is also regulated by c-di-GMP and promotes attachment and biofilm formation in the plant pathogen *Agrobacterium tumefaciens* [37,51]. The *R. etli* double mutant impaired in the production of both cellulose and MLG β-glucans (Cel^−^ MLG^−^) but was still able to attach to roots at high rates (Figure 5B), which suggests that other EPS, such as UPP, might be important for Ret attachment to the plant roots. 

Regarding symbiosis, Cel^−^ mutants of *R. etli* formed wild-type symbiosis with common bean plants, indicating that cellulose does not appear to be important for nodulation or nitrogen fixation. This is in agreement with previous research reporting that *R. leguminosarum* bv. viciae and *R. leguminosarum* bv. trifolii cellulose-negative mutants do not show nodulation defects [49,50]. As discussed by these authors, these results do not exclude the possibility that cellulose can have a more important role for nodulation under field conditions, for instance by contributing to nodulation competitiveness. On the contrary, common bean plants inoculated with MLG^−^ mutants showed slightly reduced shoot and nodule biomasses. This statistically non-significant trend, however, resulted in a slight but statistically significant reduction in the shoot N contents of the plants (Figure S3). This suggests that MLG could contribute to the fitness of interaction between *R. etli* and its host.

A much different situation is found when β-glucans are overproduced in the presence of the DGC PleD*. We have previously reported that high c-di-GMP contents are detrimental to nodulation and nitrogen fixation by *R. etli* [19]. Here we show that the overproduction of cellulose, alone but specially in combination with MLG overproduction, partially contributes to this deleterious effect of c-di-GMP on the symbiotic efficiency (Figure 6 and Figure S3). The Cel^−^ and the Cel^−^ MLG^−^ mutants harboring PleD* formed more efficient symbioses than the wild-type expressing PleD*. No significant alterations in the nodule structure were observed (Figure S4). However, it is not unreasonable to imagine that although cellulose can have a positive role during the early steps of this plant–bacteria interaction, a continuous overproduction of this β-glucan over the whole infection process, e.g., through the infection thread, could have a pernicious effect for the symbiotic establishment. This seems also to be the case in other bacteria-host associations, where the overproduction of cellulose generated by high c-di-GMP intracellular levels negatively affects the production of other EPS involved in the symbiotic process [52].

Nevertheless, the double mutant strain (Cel^−^ MLG^−^) in presence of pleD* still displayed a reduced symbiotic efficiency compared to the wild type. The high c-di-GMP contents in this double mutant determined a reduction of the N content of the host plants (Figure S3). This indicates that besides cellulose and MLG production, additional c-di-GMP-regulated molecules or processes that are important for symbiotic establishment, are likely deregulated by the expression of the DGC PleD*. Future studies will try to identify these other c-di-GMP targets and their contribution to the dual lifestyles of rhizobia.

## 5. Conclusions

In this work, we described *Rhizobium etli* as the first bacterium reported to be able to produce cellulose and MLG.

The production of both β-glucans is negligible under laboratory conditions and was triggered by increasing the intracellular levels of c-di-GMP. This c-di-GMP activation seems to occur at the post-translational level.

Under a c-di-GMP-activated state, there is an interplay among cellulose and MLG production in *Rhizobium etli* since the loss of one of these β-glucans favors the production of the other.

Cellulose and MLG contribute differentially to the bacterial phenotypes analyzed. Cellulose production promoted by PleD* favored the early stages of the interaction with the host plant, similar to biofilm formation and plant root colonization, but resulted in a decreased symbiotic efficiency. Similar to cellulose, MLG is not essential to establish effective symbiosis, albeit it could help *Rhizobium etli* to achieve the highest efficiency levels during its symbiotic interaction with bean plants.

## Data Availability

Not applicable.

## Supplementary Materials

The following supporting information can be downloaded at: https://www.mdpi.com/article/10.3390/biology11091364/s1, Figure S1: Neighbor-joining phylogenetic analysis of all the glycosyltransferases (GT) of *R. etli* CFN42 according to carbohydrate-active enzyme database (CAZY); Figure S2: Impact of c-di-GMP on the regulation of MLG production in *R. etli* CFN42; Figure S3: Role of β-glucans Cellulose and MLG in the symbiotic interaction of *R. etli* CFN42 with bean plants; Figure S4: Structure of *Phaseolus vulgaris* nodules induced by different *R. etli* strains; Table S1: Primers used in this work.
