# Supplementary material for: The Role of Two Linear β-Glucans Activated by c-di-GMP in Rhizobium etli CFN42
Source: Biology (Basel). 2022 Sep 17;11(9):1364. doi: 10.3390/biology11091364 (PMC9495663; doi:10.3390/biology11091364)
Supplement: Supplementary file 1 [file biology-11-01364-s001.zip › biology-1902660-supplementary.pdf]

biology

MDPI

Supplementary Material

Role of two linear β-glucans activated by c-di-GMP in *Rhizobium etli* CFN42

Daniel Pérez-Mendoza, Lorena Romero-Jiménez, Miguel Ángel Rodríguez-Carvajal, María J. Lorite, Socorro Muñoz, Adela Olmedilla and Juan Sanjuán

Table S1. Primers used in this work.

Primer	Product (bp)	Sequence (5′ to 3′)	Used in
362-F	114	GGAAGAACCGCTCAAGG	qRT-PCR
362-R		GTGTTGCGATCTGCCTC	
363-F	127	TTTCTGATATTGATGAGCCG	qRT-PCR
362-R		GCTCGTTATAGGTGGGAATG	
363-1	939	CGACGGACAGAATGACG	Deletion of *bgsA*
363-2		TCGTTGGGTCAGTTCACGCTTGTCGC	
363-3	808	CGTGAACTGACCCAACGACGAAGCAG	
363-4		TACGGTTGCTGCGACAC	

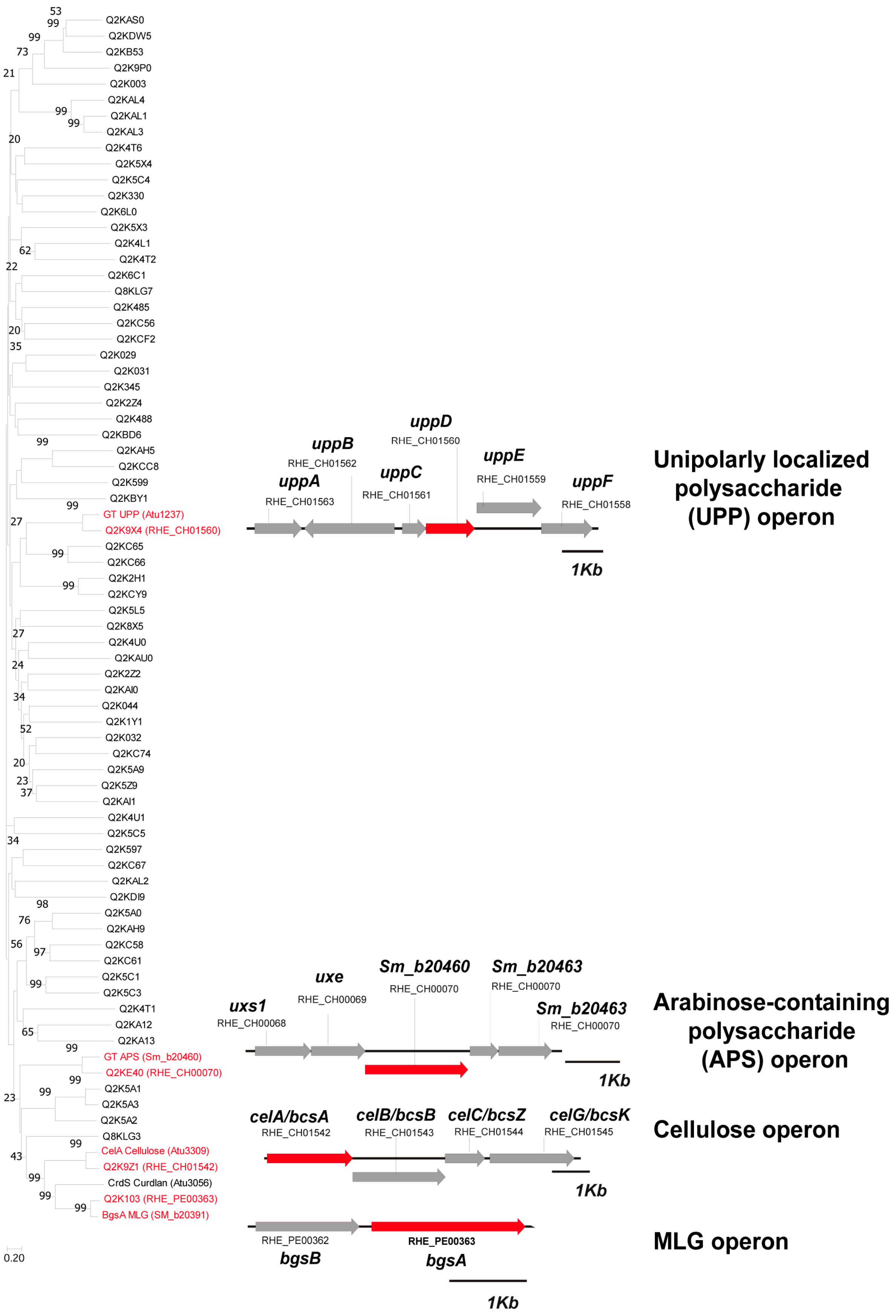

Figure S1. Neighbor-joining phylogenetic analysis of all the glycosyltransferases (GT) of *R. etli* CFN42 according to carbohydrate-active enzyme database (CAZY) ([32]; www.cazy.org) plus other GTs reported to be involved in the synthesis of other c-di-GMP regulated EPS from different

Rhizobiaceae strains: Cellulose (CelA; Atu3309), Curdlan (CrdS; Atu3056) and UPP (Atu1237) from *A. tumefaciens* C58 and MLG (BgsA; SM_b20391) and APS (SM_b20460) from *S. meliloti* 8530. Proteins are represented by their Uniprot reference number. Bootstrap values (500 replicates) are indicated at branching points. Schematic diagrams of ORFs in representative operons are shown by red arrows for GTs and gray arrows for others.

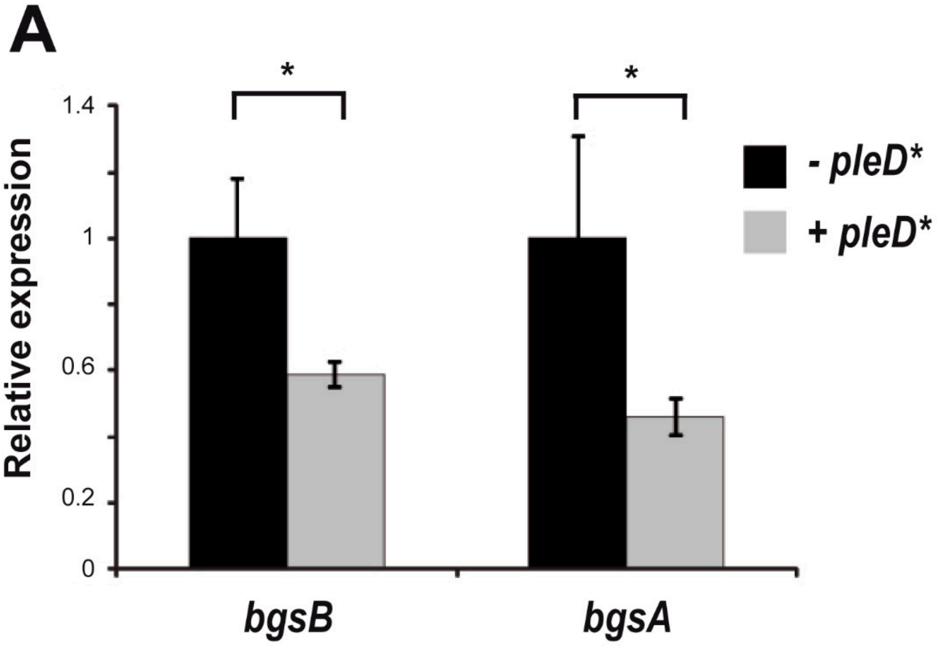

B

```
                         1                                                50
C-ter Ret BgsA     (1)  PVAAWWSGINIVVLLIASLICFEKPRRLFHAFKLDEPAVVDDVPGQIVSL
C-ter Sme BgsA     (1)  AIAALWSGINIVVLIIASLICFEKPRRLLQAFKLDEPVDVDGVEGRLVSL
     Consensus     (1)   IAA WSGINIVVLIIASLICFEKPRRL  AFKLDEP  VD V G IVSL
                         51                                              100
C-ter Ret BgsA    (51)  ALDKAVVAVHNMARFQSKSVMLKIAGFDPIKAELGQVTQRRSSISRGGDR
C-ter Sme BgsA    (51)  SLDKAVVAVSTETRFKSTKVRLNIEGFAPLEADLKQVTQRRGDITRTGDK
     Consensus    (51)  ALDKAVVAV    RF S  V L I GF PI ADL QVTQRR  ISR GDK
                         101                                             150
C-ter Ret BgsA   (101)  QAYYLHLYFELSGAARDSMIVKLYTGQYSRDIRDIDKVAVSLNLLLRSFG
C-ter Sme BgsA   (101)  QRYYLHLHYDLRGVERDKMIIKLYTGRYSRDVPDIDKIAVSVNLLLRAFG
     Consensus   (101)  Q YYLHLHFDL G  RD MIIKLYTG YSRDI DIDKIAVSLNLLLRAFG
                         151
C-ter Ret BgsA   (151)  RTRTL
C-ter Sme BgsA   (151)  RTRTA
     Consensus   (151)  RTRT
```

Figure S2. Impact of c-di-GMP on the regulation of MLG production in *R. etli* CFN42 **(A)** Relative expression of MLG biosynthetic genes *bgsB* and *bgsA* in the presence (grey) and absence (black) of *pleD**. Mini-Tn7 strains with and without *pleD** were grown in TY at 28 ºC until reaching an OD_{600nm} of 0.8-0.9, after which the mRNA was extracted. Bars represent the mean of three independent biological replicates with three technical replicates ± the standard error. Expression was normalized with the 16S gene. Asterisks indicate significant differences using an analysis of variance with a level of significance $p<0.05$ **(B)** Alignment of the c-terminal domain of BgsA from *R. etli* CFN42 (Ret) and *S. meliloti* 8530 (Sme). Color codes: white on black background = invariant residues and black on white = non-conserved. On the consensus sequence, residues required for c-di-GMP binding in $BgsA_{sme}$ are bold faced and those affected in the c-di-GMP binding are underlined [17].

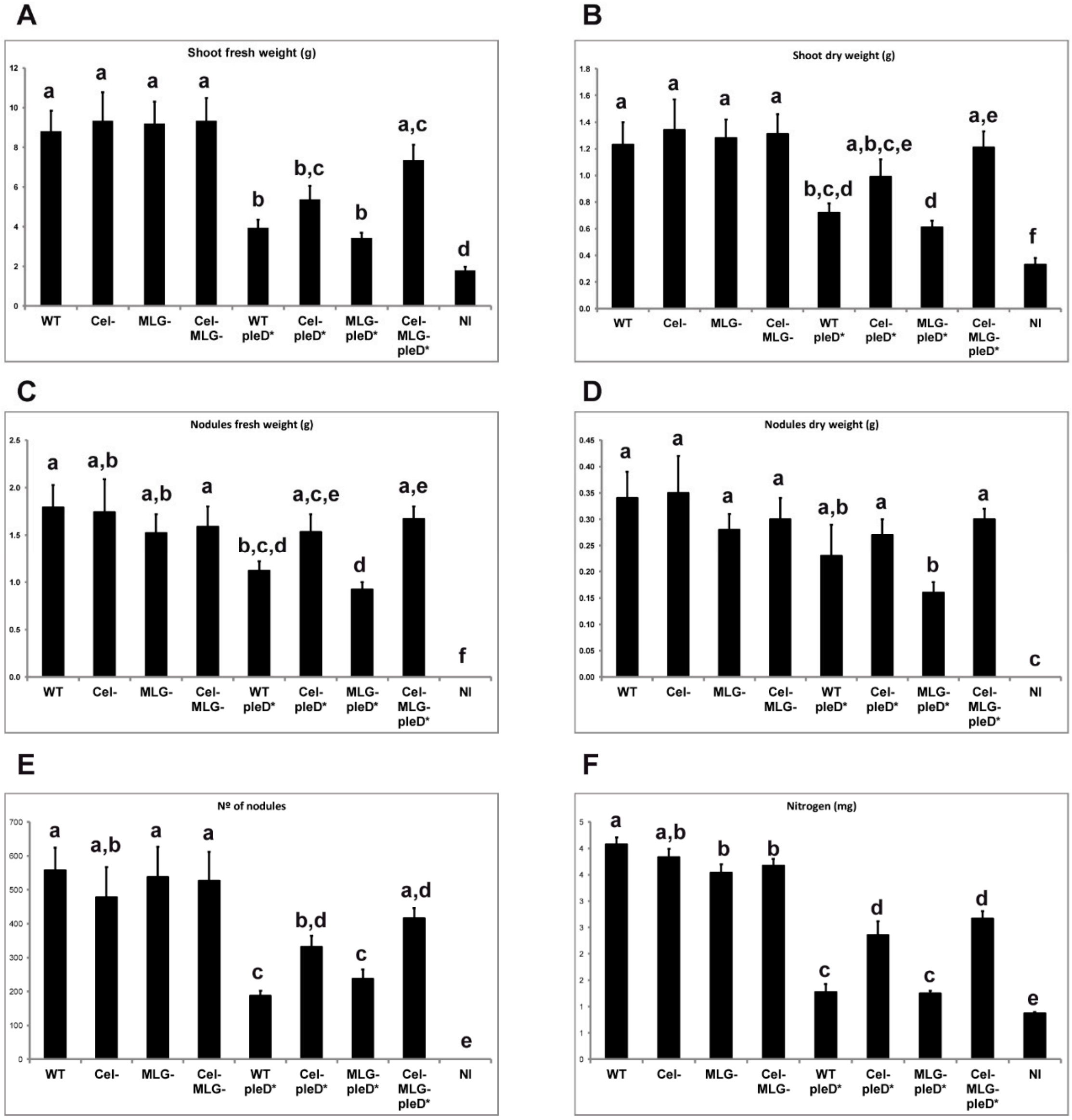

Figure S3. Role of β-glucans cellulose and MLG in the symbiotic interaction of *R. etli* CFN42 with bean plants. The bars indicate the mean±SEM of weight in grams (g) of plant shoots fresh **(A)** and dry **(B)**, the weight of nodules fresh **(C)** and dry **(D)**, the number of nodules per plant **(E)** and the nitrogen content of shoots **(F)** in milligrams (mg) of 29 days' plants inoculated with single mutants in *celAB* cellulose genes (Cel^-), in *bgsA* (MLG^-), the double mutant (Cel^- MLG^-) and their respective parental strains (WT) with or without *pleD** plus the non-inoculated (NI). Different letters over the bars indicate statistical significant differences with a $p<0.05$.

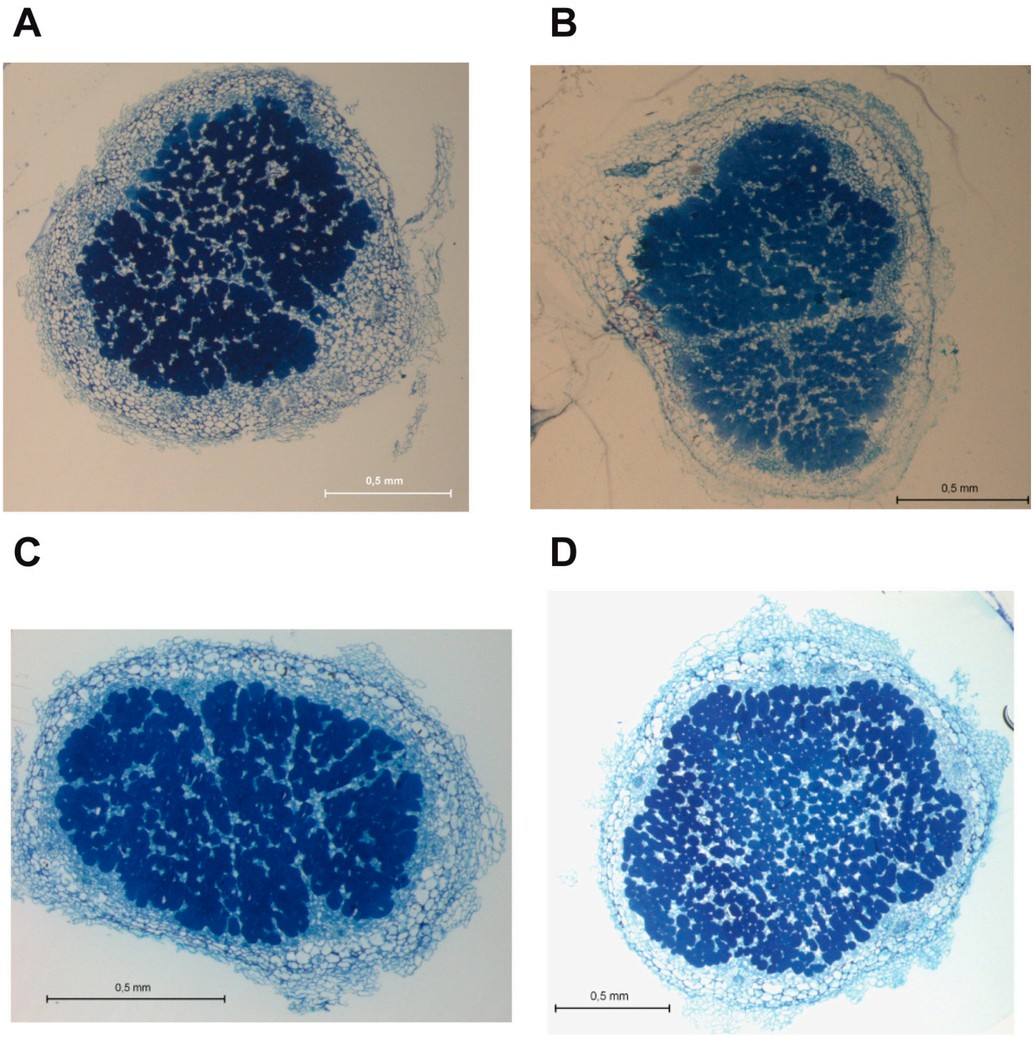

Figure S4. Structure of *Phaseolus vulgaris* nodules induced by different *R. etli* strains. Representative semithin (1 µm) sections of nodules infected by *R. etli* Tn7Km **(A)**, Ret Tn7pleD*Km **(B)**, Ret Cel⁻ MLG⁻ Tn7Km **(C)** and Ret Cel⁻ MLG⁻ Tn7pleD*Km **(D)**. Sections for light microscopy were stained with 1% (w/v) toluidine blue in aqueous 1% sodium borate for direct observation with a Zeiss Axioskope photomicroscope. Scale bars of 500 µm are depicted.

References

17. Pérez-Mendoza, D.; Bertinetti, D.; Lorenz, R.; Gallegos, M.T.; Herberg, F.W.; Sanjuan, J. A novel c-di-GMP binding domain in glycosyltransferase BgsA is responsible for the synthesis of a mixed-linkage beta-glucan. *Sci Rep* **2017**, *7*, 8997, doi:10.1038/s41598-017-09290-2.

32. Drula, E.; Garron, M.-L.; Dogan, S.; Lombard, V.; Henrissat, B.; Terrapon, N. The carbohydrate-active enzyme database: functions and literature. *Nucleic Acids Research* **2021**, *50*, D571-D577, doi:10.1093/nar/gkab1045.
